# Second-generation compound for the modulation of utrophin in the therapy of DMD

**DOI:** 10.1093/hmg/ddv154

**Published:** 2015-05-01

**Authors:** Simon Guiraud, Sarah E. Squire, Benjamin Edwards, Huijia Chen, David T. Burns, Nandini Shah, Arran Babbs, Stephen G. Davies, Graham M. Wynne, Angela J. Russell, David Elsey, Francis X. Wilson, Jon M. Tinsley, Kay E. Davies

**Affiliations:** 1 Medical Research Council Functional Genomics Unit, Department of Physiology, Anatomy and Genetics, University of Oxford, Oxford OX1 3PT, UK,; 2 Department of Chemistry, University of Oxford, 12 Mansfield Road, Oxford OX1 3TA, UK,; 3 Department of Pharmacology, University of Oxford, Mansfield Road, Oxford OX1 3PT, UK and; 4 Summit Therapeutics plc, 85b Park Drive, Milton Park, Abingdon, OxfordshireOX14 4RY, UK

## Abstract

Duchenne muscular dystrophy (DMD) is a lethal, X-linked muscle-wasting disease caused by lack of the cytoskeletal protein dystrophin. There is currently no cure for DMD although various promising approaches are progressing through human clinical trials. By pharmacologically modulating the expression of the dystrophin-related protein utrophin, we have previously demonstrated in dystrophin-deficient *mdx* studies, daily SMT C1100 treatment significantly reduced muscle degeneration leading to improved muscle function. This manuscript describes the significant disease modifying benefits associated with daily dosing of SMT022357, a second-generation compound in this drug series with improved physicochemical properties and a more robust metabolism profile. These studies in the *mdx* mouse demonstrate that oral administration of SMT022357 leads to increased utrophin expression in skeletal, respiratory and cardiac muscles. Significantly, utrophin expression is localized along the length of the muscle fibre, not just at the synapse, and is fibre-type independent, suggesting that drug treatment is modulating utrophin transcription in extra-synaptic myonuclei. This results in improved sarcolemmal stability and prevents dystrophic pathology through a significant reduction of regeneration, necrosis and fibrosis. All these improvements combine to protect the *mdx* muscle from contraction induced damage and enhance physiological function. This detailed evaluation of the SMT C1100 drug series strongly endorses the therapeutic potential of utrophin modulation as a disease modifying therapeutic strategy for all DMD patients irrespective of their dystrophin mutation.

## Introduction

Duchenne muscular dystrophy (DMD) is an X-linked recessive disorder caused by genetic mutation in the dystrophin gene and characterized by progressive muscle wasting and weakness (1). This disorder affects 1 in 3500 boys and *de novo* mutations continue to arise in all populations worldwide (2). Males who carry the dystrophin mutations display normal development until 3–5 years of age, after which the first signs of DMD manifest as abnormal gait, weakness in proximal muscles and calf muscle pseudo hypertrophy. These symptoms progress relentlessly and patients usually require wheelchair support by the age of 12 years (3,4) and succumb to heart or respiratory failure by 30 years of age (5).

Dystrophin is essential to maintain the biomechanical properties of fibre strength, flexibility and stability in skeletal muscle. It forms part of the dystrophin-associated protein complex (DAPC) which comprises many other proteins including dystroglycans, sarcoglycans, α-dystrobrevin, syntrophins and sarcospan. This complex assembles at the sarcolemma to form a stable link between the extracellular matrix and actin cytoskeleton allowing myofibres to cope with repeated cycles of muscle contraction and relaxation (6). DMD patients experience repeated cycles of muscle necrosis and regeneration leading to eventual replacement of muscle fibres by adipose and connective tissue (7). The urgency to seek a cure for DMD has resulted in parallel efforts to develop exon skipping (8,9), termination codon read through (10), dystrophin gene replacement or editing therapies (11,12) and development of non-dystrophin strategies (13–15). Each strategy has its potential caveats and may not benefit all DMD patients.

Utrophin is a structural and functional autosomal paralogue of dystrophin (16) that is ubiquitously localized at the sarcolemma *in utero* and is progressively replaced by dystrophin (17–19). In adults, the utrophin A isoform is enriched at the neuromuscular and myotendinous junctions of skeletal muscle (20) as well as the sarcolemma of regenerated myofibres (21). In DMD patients and the *mdx* mouse model, utrophin is naturally increased in regions of the fibres undergoing repair due to the absence of dystrophin (21,22). Studies with a transgenic *mdx* mouse expressing utrophin under the control of the HSA promoter (*Fiona mice)* have shown that in muscle, a 3–4 fold increase in wild-type utrophin protein levels successfully prevents the dystrophic pathology (23,24). Importantly, this utrophin protein increase is significantly less than the normal utrophin protein levels in kidney and liver (23). Deliberately over expressing utrophin protein showed no detrimental effect in a broad range of murine tissues (25).

Despite its functional similarities to dystrophin, utrophin exhibits different modes of interaction with actin (26) and microtubules, and may not prevent microtubule lattice derangement (27). It is important to note that the muscle function is fully restored in the *Fiona* mice (28), suggesting that microtubule arrangement is likely to be part of a more complex mechanism of contraction-induced injury in the *mdx* mouse and probably not the sole contributing factor involved in this phenomenon. Unlike dystrophin, utrophin is unable to restore nNOS localization (29). However, a recent study reported no relationship between the expression of nNOS at the sarcolemma and the disease severity in Becker patients (30) as many BMD patients lacking the nNOS binding site in dystrophin remain mildly affected and ambulant. The constitutively expressing utrophin *mdx* mouse showed significant improvement without nNOS membrane localization, suggesting that there may be compensatory nNOS pathways (29,31). Despite these subtle differences between utrophin and dystrophin, a small increase in utrophin levels delays the age of wheelchair support in patients (32) and utrophin can act as an effective surrogate for dystrophin in *mdx* muscles (24,33,34).

The significant advantage of utrophin modulation therapy and the continual expression of utrophin in muscle and heart is that the approach is applicable to all DMD patients regardless of the dystrophin mutation. Furthermore, a systemic strategy designed to increase the endogenous utrophin level to treat all skeletal muscle (including the diaphragm) and heart, would not be anticipated to result in an immune response (25). To date, direct delivery of the protein (35) or stabilization of the protein or RNA (36,37), viral approaches (38,39), non-viral strategies such as recombinant biglycan (40) and oral compound administration designed to modulate the utrophin expression at the transcriptional level (41,42), showed that maintaining utrophin expression in *mdx* muscle fibres could decrease the progression of the disease and represents a promising therapeutic avenue for DMD.

SMT C1100 was the first new chemical entity (NCE) specifically designed to target the utrophin-A promoter (43) which has progressed into clinical development. *Mdx* mice showed marked improvement in functional calcium-dependent parameters, decreased serum creatine kinase levels, muscle fibrosis, necrosis and membrane damage in skeletal muscle following eccentric contractions (41). SMT C1100 was deemed safe and well tolerated in a Phase 1a healthy volunteer study, although with a lower than expected drug exposure level (15) and successfully completed a Phase 1b study in DMD boys.

In this study, we evaluated in greater depth another compound, SMT022357, from the SMT C1100 family, for its effects on several skeletal muscle groups, including the diaphragm, and heart. SMT022357 is a second-generation compound structurally related to SMT C1100 with improved physicochemical properties and a more robust metabolism profile. This compound showed effective up-regulation of utrophin in all muscle groups and significant improvement in the overall muscle pathophysiology of the *mdx* mouse. These results further cement the rationale of developing novel NCEs capable of modulating utrophin transcription as a potential therapy for all DMD patients.

## Results

### *In vitro* upregulation of utrophin

To study the ability of SMT022357 to increase the transcription of the endogenous mouse utrophin gene (*Utrn*), we used the screening line H2K-*mdx* utrnA-luc (41). These murine myoblast cells contain a stably integrated 8.4 kb of the human utrophin promoter linked to a luciferase reporter gene. This region of the utrophin promoter includes all the motifs known to control utrophin expression as previously described (44).

**Figure 1.**
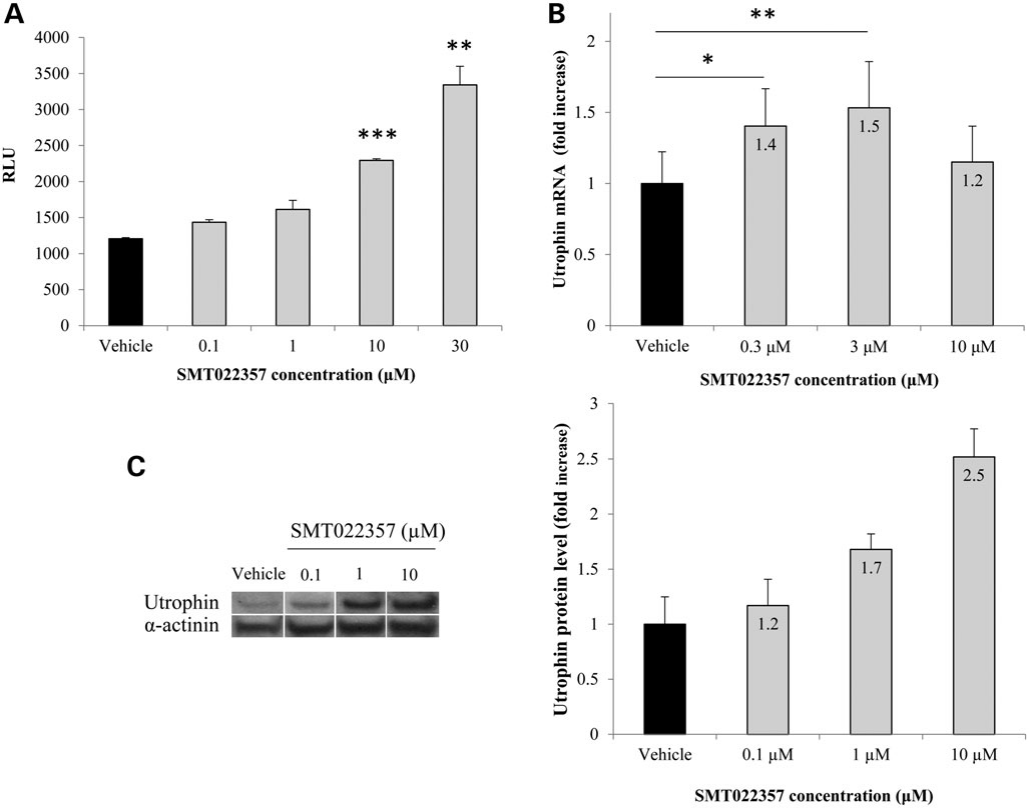
*In vitro* activity of SMT022357. (**A**) SMT022357 *in vitro* dose response in murine H2k-*mdx* utrnA-luc cells expressing the human utrophin promoter linked to a firefly luciferase reporter gene. Cells were treated with compound for 24 h in standard growth medium containing 1% DMSO. The chart shows relative luminescence (RLU) in relation to five different doses (0.1, 0.3, 1, 3 and 10 µM) of SMT022357. (**B**) SMT022357 significantly increased mRNA copy number of the utrophin transcript in murine myoblast cells. Cells were exposed to different dose of SMT022357 in standard media with 0.1% DMSO (vehicle) for 24 hours with six biological replicates. Utrophin transcripts were normalised with *18s* and *b2m*. Values are mean ± SEM of *n* = 6 per condition; *P < 0.05, **P < 0.01, ***P < 0.001. (**C**) Relative utrophin protein expression in murine cells treated with SMT022357 was determined by western blot and standardized for α-actinin loading. Western blots showing 2.5-fold increase of utrophin protein expression with 10 µM of SMT022357 when compared with vehicle. Relative utrophin expression is shown as mean ± SEM of *n* = 3 per condition.

SMT022357 shows a maximal increase of 3-fold in luciferase compared with vehicle (Fig. 1A). No stabilization or inhibition of the luciferase activity was noted (data not shown). Furthermore, no change in proliferation was observed after a treatment with SMT022357 (Supplementary Material, Fig. S1). *In vitro* dosing of murine myoblasts with 3 µm of SMT022357 led to a 50% increase in utrophin mRNA when compared with vehicle after 2 days of treatment (Fig. 1B). Treatment of murine DMD cells with SMT022357 showed a 2.5-fold increase in utrophin protein level at an optimal concentration of 10 µm after 3 days of treatment (Fig. 1C). In comparison, SMT C1100 led to a 30% increase in *Utrn* mRNA level and resulted in a 2.0-fold increase in UTRN protein level (41). These data demonstrate the *in vitro* potential of SMT022357 to increase the level of utrophin expression.

### *In vivo* upregulation of utrophin

**Figure 2.**
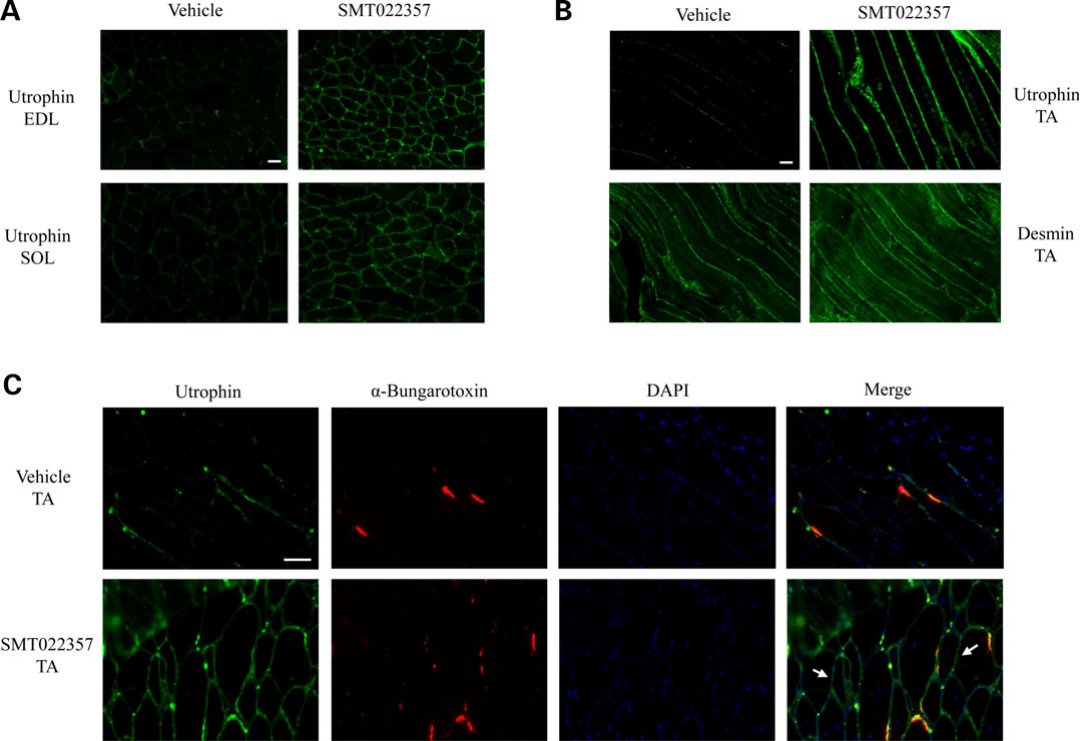
SMT022357 increases utrophin expression in skeletal muscles. (**A**) Immunofluoresence staining for utrophin in EDL and SOL muscles of 7 weeks old *mdx* mice treated for 5 weeks with 30 mg/kg/day SMT022357 or vehicle. Transverse sections were stained with anti-utrophin polyclonal antibody URD40 and anti-rabbit secondary antibody. Scale bar: 100 µm. (**B**) Longitudinal cryosections of TA muscle following treatment of *mdx* mice homogenously increased the utrophin expression along the myofiber after treatment with SMT022357 compared to vehicle group. Scale bar: 100 µm. (**C**) Co-immunohistochemical staining of TA muscle with utrophin, α-Bungarotoxin and DAPI prepared from *mdx* mice treated with SMT022357. White arrows indicate utrophin expression in extra-synaptic regions of the sarcolemma. Scale bar: 50 µm.

To investigate the *in vivo* activity of SMT022357, 2-week-old *mdx* mice were orally dosed daily with 30 mg/kg of SMT022357 or vehicle for 5 weeks. Immunofluorescence using a utrophin antibody revealed a qualitative increase in sarcolemma-bound utrophin in the transverse of EDL and SOL muscles (Fig. 2A). In the *mdx* mouse, utrophin localization is fragmented and discontinuous along the myofibre highlighting the regions within the fibre where regeneration is taking place in order to repair the damaged membrane (Fig. 2A–C). After treatment with SMT022357, utrophin was continuously localized along the entire length of the sarcolemma in longitudinal tibialis anterior (TA) sections suggesting that drug treatment is modulating utrophin transcription in myonuclei along the length of the fibre (Fig. 2B). The integrity of the membranes was confirmed with desmin immunostaining (Fig. 2B). Co-localization with α-bungarotoxin antibody in TA muscles confirmed, despite a preferred accumulation at the neuromuscular junction, a qualitative increase of utrophin at extra-synaptic regions of the sarcolemma (Fig. 2C). Western blot analysis and quantification in EDL and SOL muscles demonstrated a 1.8- and 1.9-fold increase, respectively, of the utrophin protein levels after treatment with SMT022357 (Supplementary Material, Fig. S2A).

**Figure 3.**
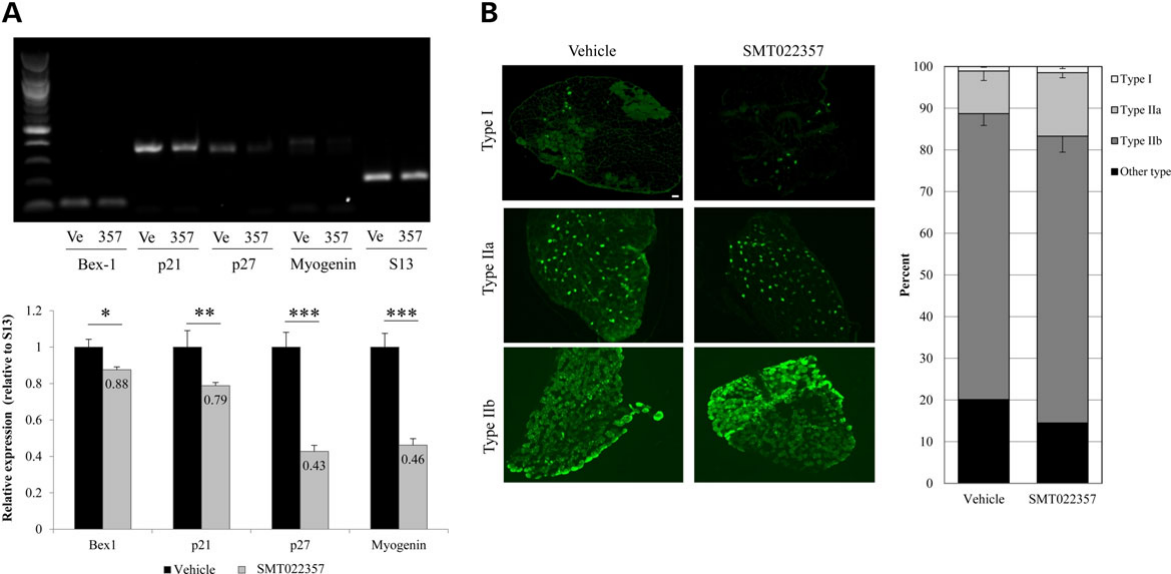
SMT022357 treatment results in a decrease in regeneration with no change in fibre type composition in the skeletal muscle. (**A**) Semi-quantitative RT-PCR demonstrates a decrease of skeletal muscle regeneration/myogenic differentiation markers Bex1, myogenin and cell cycle dependent-kinase inhibitors (CDKI) p21 and p27 from EDL of *mdx* mice dosed with SMT022357 (357, *n* = 8) compared to vehicle (Ve, *n* = 8). S13 ribosomal protein was used as an internal control for the RT-PCR. A 12% decrease in Bex1 (*P* = 0.034), 21% decrease in p21 (*P* = 0.002), 67% decrease in p27 (*P* < 0.001) and 64% decrease in Myogenin (*P* < 0.001) transcript levels were quantified by ImageJ software in SMT022357 group compared to vehicle. (**B**) Immunofluoresence staining for type 1a, 2a and 2b fibre types in the EDL of 7 weeks old *mdx* mice treated for 5 weeks with 30 mg/kg/day SMT022357 or vehicle (*n* = 6 per group). Sections were stained with anti-MYHC1, anti-MYHC2A anti-MYHC2B and anti-mouse secondary antibody. Percentage of type 1a, 2a and 2b fibre types was quantified by ImageJ software in the whole EDL muscle (*n* = 6 per group). No change was observed in fibre type composition of whole EDL muscle treated with SMT022357 compared to treatment with vehicle only. Scale bar: 100 µm.

Utrophin is up-regulated during regeneration in skeletal muscle (21). In order to study regeneration after treatment with SMT022357, the expression of different regeneration/myogenic differentiation markers (45) was analysed by semi-quantitative RT–PCR. A decrease in all these markers was noted in EDL muscles treated with SMT022357 (Fig. 3A), demonstrating that daily drug treatment for 5 weeks results in a reduction in regeneration and, importantly, that the increase of utrophin is independent of this process. This suggests that the higher levels of utrophin must be derived from drug treatment rather than regeneration. It is also well established that dystrophic muscle type I fibres present greater utrophin expression than type II fibres (36,46,47). Thus, we investigated the expression of type I, IIa and IIb myosin heavy chains in EDL muscle. No change in fibre-type composition was noted after treatment with SMT022357 (Fig. 3B), supporting the fact that SMT022357 increases the utrophin expression in both fast-twitch (type II) and slow-twitch (type I) fibres.

Taken together, our results demonstrated that daily oral treatment with SMT022357 for 5 weeks in the *mdx* mouse resulted in increased levels of utrophin throughout the dystrophic myofibres and extrasynaptic domains, independently of regeneration or change in fibre-type composition.

### SMT022357 ameliorates the pathology in skeletal muscle

The *mdx* mouse exhibits a mild form of muscular dystrophy with elevated serum levels of creatine kinase (48) and histological changes consistent with myofibre damage (49). Treatment with SMT022357 results in a 47% decrease of serum CK levels (Fig. 5A), demonstrating that the increased levels of utrophin derived from drug treatment is reducing the amount of membrane damage reducing the rate of CK leaking into general circulation.

**Figure 4.**
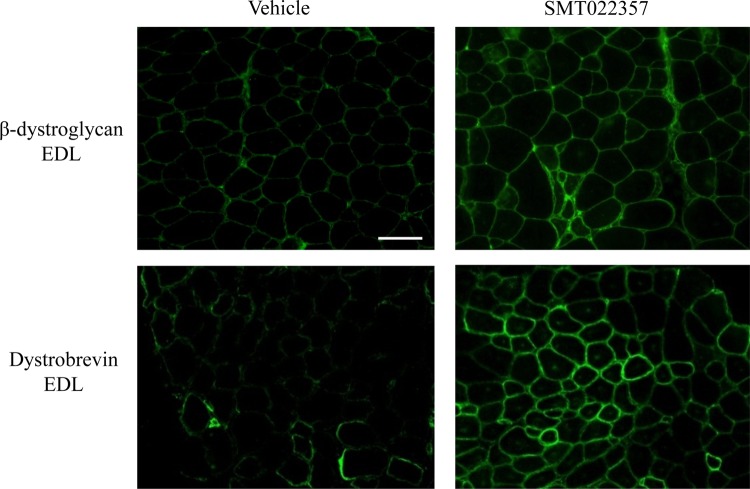
SMT022357 treatment improves fibre membrane stability. Immunofluoresence staining for β-dystroglycan, and dystrobrevin in EDL muscle of 7 weeks old *mdx* mice treated for 5 weeks with 30 mg/kg/day SMT022357 or vehicle shows that key components of the DAPC complex are properly localised to the sarcolemma after SMT022357 treatment. *n* = 3 per group; Scale bar: 100 µm.

Next, we evaluated the restoration of membrane stability in dystrophin-negative myofibres following the increased level of utrophin at the sarcolemma. Key elements of the DAPC complex such as β-dystroglycan, which directly binds to laminin in the extracellular matrix, are not properly localized into the sarcolemma in DMD patients resulting in muscle damage after repeated contractions (50). As illustrated in Figure 4, in EDL muscles treated with SMT022357, localization of β-dystroglycan and dystrobrevin (another member of the DAPC) was restored to the sarcolemma of all fibres. This translated to a 1.7- and 1.3-fold increase of β-dystroglycan and dystrobrevin expression, respectively, when quantified by western blots (Supplementary Material, Fig. S2B). This data predicts an improvement of muscle membrane stability when utrophin acts as a dystrophin surrogate to maintain the DAPC complex including the major component of the DAPC-laminin axis.

**Figure 5.**
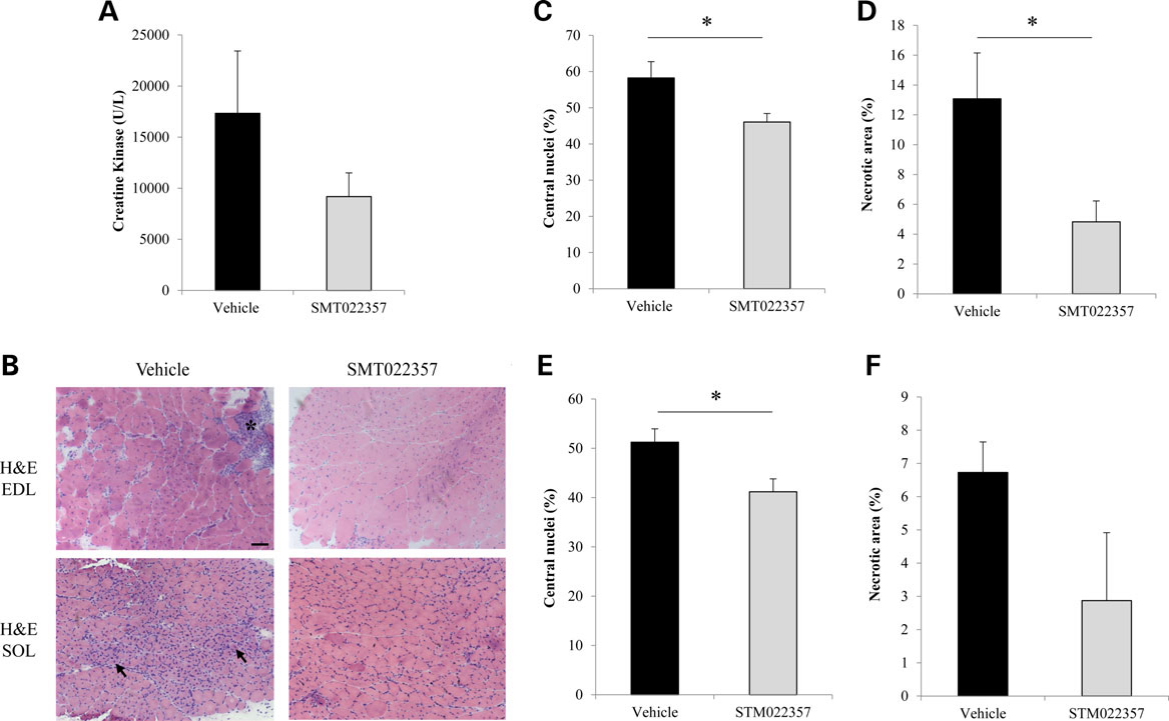
SMT022357 prevents muscular dystrophy in skeletal muscle. (**A**) Creatine kinase (CK) levels in serum following daily oral gavage of *mdx* mice with 30 mg/kg of SMT022357 or vehicle from two weeks of age for five weeks. A 47% decrease in serum CK was observed after treatment with SMT022357 compared to vehicle-treated animals (*n* = 7-9 per group). (**B**) H&E-stained transverse muscle sections of EDL muscle (7 weeks of age) in untreated *vs.* treated *mdx* mice (*n* = 10 per group) showing necrotic areas (black stars) and regenerating fibres (black arrows). Scale bar: 100 µm. (**C**) Muscle from mice dosed with SMT022357 showed a significant 20.1% (*P* = 0.03) decrease in centrally nucleated fibres compared to the vehicle group in EDL muscles. Values are mean ± SEM of *n* = 9 per groups; *P < 0.05. (**D**) The necrotic muscle area in EDL of mice treated with SMT022357 significantly decreased by 63.1% (*P* = 0.02) compared to the vehicle group. Values are mean ± SEM of *n* = 9 per groups; *P < 0.05. (**E**) Muscle from mice dosed with SMT022357 showed a significant 19.6% (*P* = 0.02) decrease in centrally nucleated fibres compared to the vehicle group in SOL muscles. Values are mean ± SEM of *n* = 7 per groups; *P < 0.05. (**F**) The necrotic muscle area in SOL of mice treated with SMT022357 decreased by 57.3% (*P* = 0.06) compared to the vehicle group. Values are mean ± SEM of *n* = 7 per groups.

Improved muscle pathology of SMT022357 treated *mdx* mice compared with vehicle was observed in SOL (type I) and EDL (type IIb) muscles (Fig. 5B). A biomarker of fibre regeneration, the number of centrally nucleated fibres, fell significantly in EDL (−20.1%, *P* = 0.03, Fig. 5C) and SOL (−19.6%, *P* = 0.02; Fig. 5E) resulting in a reduction of the necrotic areas in EDL (−63.1%, *P* = 0.02, Fig. 5D) and SOL (−57.3%, *P* = 0.06. Fig. 5F). This clearly demonstrates a significant reduction in pathology and regeneration in these muscles. The 2-week-old *mdx* mice present a 18.3% of centronucleation (SEM = 0.01; TA, *n* = 3) and a 4.25% (SEM = 1.96; TA, *n* = 3) of necrosis compared with wild-type mouse. Therefore, SMT022357 treatment not only prevents the dystrophic pathology but may also ameliorate some parameters of the *mdx* phenotype.

**Figure 6.**
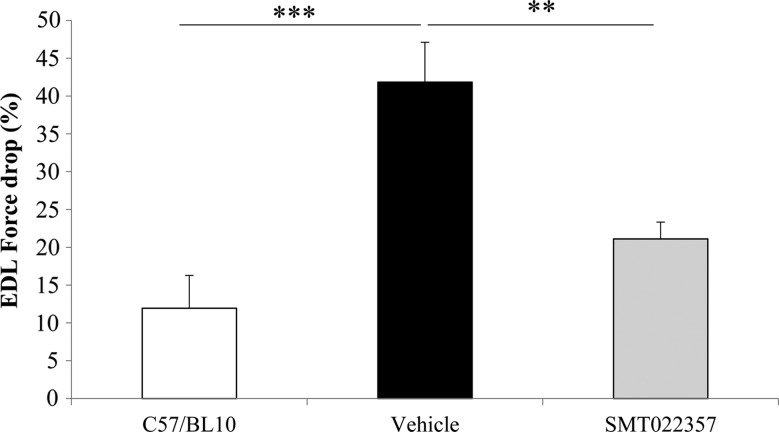
SMT022357 protects the muscle against damage and improves the muscle function. The difference in force produced between the first and fifth stretch is represented as a sensitive indicator of the resistance of the muscle to stretch-induced damage. EDL muscles were stretched at 15% of their fibre length whilst contracting tetanically. Values are mean ± SEM of *n* = 10 per group; **P < 0.01, ***P < 0.001.

Furthermore, we investigated if the increased membrane stability and reduction in pathology achieved by SMT022357 treatment translated into functional improvements in muscle. *Ex vivo* analysis of EDL muscles after 5 weeks of daily SMT022357 treatment demonstrated that after five eccentric contractions, SMT022357 results in a 50% (*P* = 0.004) decrease in force drop compared with vehicle-treated *mdx* mice (Fig. 6). No changes were observed in muscle mass, absolute or specific force when treated with SMT022357 in comparison to the vehicle (data not shown).

In summary, these data showed that daily SMT022357 treatment for 5 weeks results in increased localization of utrophin along the fibre membrane. In turn, this addresses the primary cause of fibre degeneration with the direct consequence of increased sarcolemma stability in hind-limb muscles of the *mdx* mouse, leading to favourable endpoints such as reduced regeneration and necrosis, and enhanced protection of the muscle against damage. Starting drug treatment at 2 weeks of age significantly ameliorates the pathology in the *mdx* mice. Moreover, similar benefits obtained in EDL and SOL muscle indicate that SMT022357 acts independently of the fibre-type composition, the muscle structure and the basal level of endogenous utrophin.

### Benefits of SMT022357 treatment in diaphragm and heart

In relation to DMD, it is of significant importance to confirm that the proposed therapy can reduce the dystrophin deficient defects in the diaphragm, the major respiratory muscle. Unlike skeletal and cardiac muscles, the *mdx* diaphragm closely mimics the degeneration observed in DMD patients (51–53) which suggests that the early dystrophic changes are most prominent in this muscle. In the *mdx* mouse, the diaphragm exhibits a highly dystrophic pathology and is the most reliable indicator of damage or recovery (54,55).

**Figure 7.**
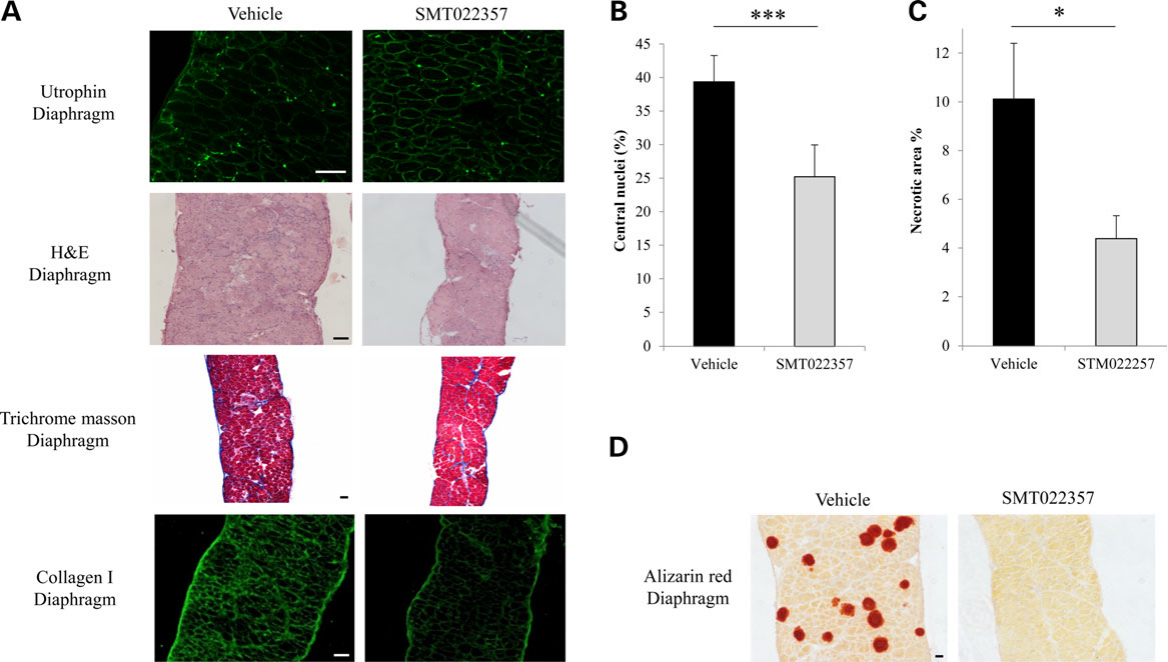
SMT022357 improves the pathology in the diaphragm. (**A**) Immunofluoresence staining for utrophin in the diaphragm of 7 weeks old *mdx* mice treated for 5 weeks with 30 mg/kg/day SMT022357 or vehicle (*n* = 10 per group). Sections were stained with anti-utrophin polyclonal antibody URD40 and anti-rabbit secondary antibody. H&E-stained transverse muscle sections of diaphragm muscle (7 weeks of age) showed improved morphology in treated *vs.* vehicle *mdx* mice (*n* = 10 per group). Masson's trichrome staining of diaphragm in untreated *vs.* treated *mdx* mice (*n* = 8) indicated that SMT022357 treatment reduced the amount of collagen infiltration (stained in blue); immunofluorescence using anti-collagen type I antibody confirmed a decrease of collagen in the muscular endomysium of SMT022357 treated diaphragm. Scale bar 100 µm. (**B**) Diaphragms of mice dosed with SMT022357 showed a significant 35.9% decrease in centrally nucleated fibres compared to the vehicle group. Values are mean ± SEM of *n* = 8 per groups; ***P < 0.001. (**C**) The necrotic diaphragm area of mice treated with SMT022357 significantly decreased by 56.6% compared to the vehicle group. Values are mean ± SEM of *n* = 8 per group; *P < 0.05. (**D**) The vehicle *mdx* diaphragm exhibits positive staining with Alizarin Red indicating the presence of calcium deposits. SMT022357 treatment completely prevented the calcification. (*n* = 10 per group); Scale bar: 100 µm.

The expression of utrophin was analysed by immunofluoresence and western blot. After treatment with SMT022357, utrophin staining appeared more homogeneous around the diaphragm fibre membranes compared with vehicle-treated mice (Fig. 7A). Western blot analysis showed that after 5 weeks of SMT022357 treatment total levels of utrophin protein were increased by 20% in the *mdx* mouse diaphragm (Supplementary Material, Fig. S2A). Analysis of the diaphragm pathology after SMT022357 treatment demonstrated a significant decrease of centrally nucleated fibres by 35.9% (*P* < 0.0001), a biomarker of regeneration, and a 56.6% (*P* = 0.04) reduction of necrotic area compared with vehicle treated (Fig. 7B and C).

In DMD muscles, endomysial fibrosis is a hallmark feature of the pathology. Although the *mdx* mouse does not recapitulate the fibrotic progression observed in patients, the *mdx* diaphragm demonstrates early dystrophic lesions including fibrosis at an early age (56). The mechanisms by which fibrosis develops in DMD patients remain largely unknown but the elevated expression of various inflammatory cytokines, such as transforming growth factor-β1 (TGF-β1), and tumour necrosis factor (TNF) stimulate an excessive amount of extracellular matrix proteins which could lead to fibrosis. Previous studies described significantly higher type-1 collagen content, TGF-β1 and MMP-2/9 mRNA levels, and signs of fibrosis in 6-week-old *mdx* mice compared with wild-type littermates (57,58). Thus, we chose to investigate the potential benefits on fibrosis development in the diaphragm of *mdx* mice treated for 5 weeks with SMT022357. Compared with mice treated with the vehicle, qualitative Trichrome Masson staining is reduced (Fig. 7A) and quantification revealed that SMT022357 resulted in a 15% decrease of fibrosis (Vehicle: fibrotic area = 6.8%, SEM = 1.03, *n* = 7; SMT022357: fibrotic area = 5.8%, SEM = 0.73, *n* = 8). Further confirmation of reduced fibrosis is seen in Figure 7A, where collagen I content is decreased in SMT022357 group.

In DMD, the lack of dystrophin leads to decreased sarcolemmal integrity resulting in an increase of cell membrane permeability leading to increased intracellular calcium. Abnormal levels of intracellular calcium leads to disruption of normal contractile signalling and activates the natural fibre repair process leading to aberrant degeneration of fibres. Of particular interest, SMT022357 treatment completely prevented the accumulation of calcium-rich deposits as depicted by Alizarin Red staining (Fig. 7D) further demonstrating the significant decrease in membrane damage. These results are in agreement with previous and recent work (24,59).

**Figure 8.**
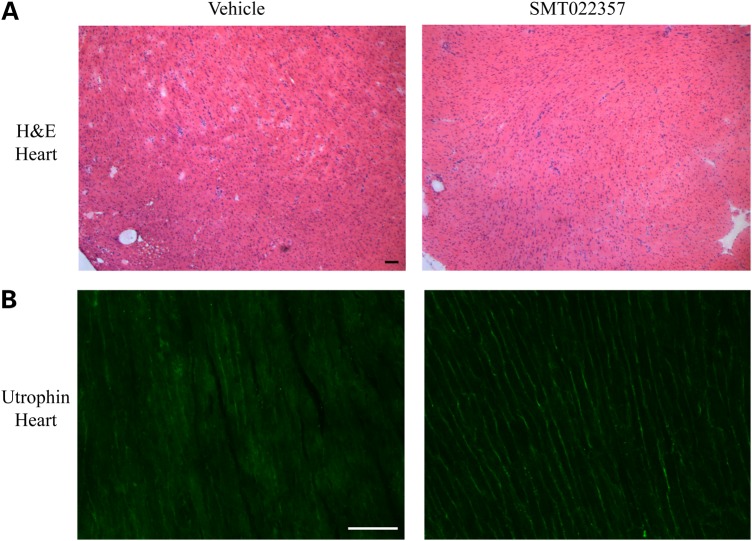
SMT022357 increases utrophin expression in the *mdx* heart. (**A**) H&E staining of transverse heart sections in 7-week-old, vehicle- and SMT022357-treated *mdx* mice. *n* = 10 per group. Scale bar: 100 µm. (**B**) Immunostaining showed an increase of utrophin in the heart of 7-week-old *mdx* mice after SMT022357 (30 mg/kg/day) treatment compared to the vehicle group. (*n* = 10 per group); Scale bar: 100 µm.

Cardiomyopathy is a major cause of death in boys with DMD (60). Preventing pathology in the heart, a muscle notoriously difficult to reach, is crucial as the benefits could lead to a significant improvement in patient survival. Western blots demonstrated a 50% increase of the utrophin protein level in the heart after 5 weeks of daily treatment with SMT022357 (Supplementary Material, Fig. S2). This increase in utrophin was wide spread throughout the heart and localized to cardiomyocyte membranes (Fig. 8). Nevertheless, analysis of 2-month-old *mdx* mouse hearts did show 1–2% fibrosis in the mid-ventricle regions and an initial increase in myocardial wall strain and torsion (61). Older 9–10-month-old *mdx* mice present distinct signs of cardiomyopathy (61,62). Further studies are on-going to determine the benefits of SMT022357 in the *mdx* heart.

## Discussion

This manuscript describes the *in vitro* and *in vivo* activity of a second-generation orally bioavailable small molecule utrophin modulator compound which targets the utrophin-A promoter. Dosing of myoblasts with SMT022357 has a similar potency to the first generation utrophin modulator SMT C1100 (41). The development of the second-generation utrophin modulators originated from a medicinal chemistry program to improve the physicochemical and metabolic properties of SMT C1100, following observations in the Phase 1 healthy volunteer trial of lower than expected exposure levels and significant SMT C1100 parent metabolism (15). SMT022357 has a more robust metabolism profile. Subsequently, the next step in the development of this second-generation molecule was to assess *in vivo* efficacy in the dystrophin deficient *mdx* mouse.

The daily oral dosing of SMT022357 in the *mdx* mouse led to increased utrophin expression in all skeletal muscles tested, diaphragm and heart, demonstrating systemic utrophin modulation and the efficient distribution of this compound. By pharmacologically modulating the expression of utrophin, SMT022357 addresses the primary cause of fibre degeneration by protecting the sarcolemma which includes maintenance of the dystrophin associated protein complex as exemplified by β-dystroglycan and dystrobrevin localization at the muscle membrane. This improvement in membrane stability leads to a significant decrease in centrally nucleated fibres (a biomarker of regeneration) and necrosis in skeletal muscles, resulting in marked functional improvement as demonstrated by the increased resistance to damage by eccentric contractions. After 5 weeks of daily oral treatment with SMT022357, utrophin is localized along the entire length of the sarcolemma. This first documented evidence of utrophin homogeneously localized along the length of 7-week-old *mdx* muscle fibres demonstrates that SMT022357 is capable of inducing and/or maintaining utrophin transcription from extrasynaptic myonuclei. The positive modulation of utrophin expression observed appears to be independent of any regeneration mechanism. Biochemical data demonstrating significant reduction in biomarkers of regeneration/myogenic differentiation, linked to significant reduction centrally nucleated fibres, confirmed reduced fibre regeneration after treatment with SMT022357. Therefore, the observation of increasing utrophin levels in muscle after drug treatment compared with the vehicle; plus the reduction in regeneration (the major source of utrophin production) suggests that dosing over the 5-week period results in significantly more fibres expressing utrophin compared with *mdx*. Furthermore, SMT022357 did not change the fibre-type composition in skeletal muscle, confirming that the modulation of the utrophin expression was independent from changes in muscle structure. Together, these data demonstrate that the increased levels of utrophin seen in skeletal and diaphragm muscle compared with vehicle-treated *mdx* mice occurs directly as a result of the treatment with SMT022357.

Interestingly, SMT022357 treatment resulted in similar increases of utrophin protein level and similar benefits in decrease of regeneration and necrosis in fibre-type I (1.9×) and fibre-type IIb (1.8×) skeletal muscles. This observation suggests that modulation of utrophin after treatment with SMT022357 is independent of the muscle structure known to influence the utrophin expression in the *mdx* mouse (47). In addition, it also suggests that SMT022357 modulates the utrophin levels by a different mechanism of action compared with AICAR, a small compound which activates PGC-1α and PPARβ/δ via AMP-activated protein kinase (AMPK), a nexus of skeletal muscle plasticity (63,64). These results support the concept that developing a multi-targeting strategy to utrophin modulation could be considered in the future.

Importantly, our results demonstrate that the oral delivery of SMT022357 distributes drug not only to skeletal muscle, but also to the diaphragm and the heart. As most DMD patients succumb to respiratory or heart failure (5,60), the potential to stabilize the disease in these organs could have additional major benefits to this treatment paradigm for DMD. After daily treatment with SMT022357 for 5 weeks, the increased level of utrophin coincides with the significantly reduced pathology of diaphragm muscle highlighted by various endpoints such as decreased regeneration, necrosis, fibrosis and calcium overload. Furthermore, in the *Fiona* mice, where utrophin is overexpressed in the skeletal muscle and the diaphragm, but only modestly in the heart, cardiac function, specifically ventricular ejection fraction, were restored to wild-type levels. Thus, ameliorating the pathology in the diaphragm muscle prevents part of the cardiac defects observed in the *mdx* mouse (65).

In muscular dystrophy, the intracellular calcium concentration is increased (66) and the excitation–contraction coupling and overall Ca^2+^ handling is impaired (67,68). Unregulated calcium influx initiates the disease pathology in dystrophin-deficient myofibres and is sufficient to cause a dystrophic phenotype in skeletal muscle independent of membrane fragility (69). Recent studies suggest that utrophin organizes local membrane microdomains containing mechanosensitive channels and that the loss of utrophin results in an increased Ca^2+^ entry (59) and previously described as one of the first defects corrected following constitutive utrophin expression (24). In addition to the recognized benefits in maintaining membrane structural integrity, utrophin may also positively act on the calcium homeostasis and further improve the excitation–contraction coupling mechanism. The complete prevention of the accumulation of calcium deposits after SMT022357 treatment further supports this theory, demonstrating the effectiveness of the utrophin transcription modulation based strategy to correct defects at an early stage of the disease.

In the *mdx* heart, SMT022357 increased the utrophin expression by 50%, confirming drug delivery to cardiac muscle. Two-month-old *mdx* mouse hearts do show evidence of cardiac lesions; first signs of fibrosis and an initial increase in myocardial wall strain and torsion (61) but only older *mdx* mice show distinct signs of cardiomyopathy (62). Further studies in older or young exercised mice (70) are needed to determine the benefits of SMT022357 treatment in the *mdx* heart. A recent publication demonstrated that increasing utrophin expression via an alternative pharmacological approach protects the *mdx* mice against cardiomyopathy (71).

Taken together, these data demonstrate that daily dosing with a second-generation utrophin modulator, SMT022357, ameliorates the dystrophic pathology in the mdx mouse. These results confirm the hypothesis that reduced fibre damage in hind-limb muscle due to an increase in utrophin levels leads to reduced regeneration and necrosis, thereby reducing muscle damage. Nevertheless, 5 weeks of treatment with SMT022357 did not normalize all the parameters studied in 7-week-old *mdx* mouse. As previously observed with the Fergie mouse, a transgenic *mdx* mouse with a mild expression of the utrophin, a 1.3/1.4-fold increase of the utrophin protein is not sufficient to obtain a complete rescue of the *mdx* phenotype (23). In future, the potential complementarity of utrophin modulation strategies with other disease stabilizing approaches such as glucocorticosteroid analogues like VBP-15 (72) or phosphodiesterase type 5 inhibitors (13) should also be explored. We have previously reported the synergistic action of SMTC1100 and prednisone (41). The significant benefit of utrophin modulation with daily oral treatment SMT022357 confirms first, that it is feasible to generate structurally different oral small molecule drugs capable of maintaining utrophin transcription and secondly, these second-generation compounds have demonstrated significant *in vivo* efficacy and better physicochemical properties compared with SMT C1100. These observations support their continued development. The data presented in this publication further endorses the therapeutic potential of utrophin modulation as a disease modifying therapeutic strategy for all DMD patients irrespective of the dystrophin mutation.

## Materials and Methods

### Ethics statement

All animal procedures were performed in accordance with UK Home Office regulations which conform with the European Community Directive published in 1986 (86/609/ EEC). The work was performed under certificate of designation number 30/2306 and project license number 30/3104 following approval by the University of Oxford Departments of Physiology, Anatomy & Genetics and Experimental Psychology Joint Departmental Ethics Review Committee.

### Cell culture

The H2K-*mdx* utrnA-luc cells (41) were maintained in DMEM (Invitrogen) supplemented with 20% fetal bovine serum gold (PAA), 5% chicken embryo extract (SLI), 2 mm l-glutamine (Invitrogen), 1% penicillin–streptomycin (Invitrogen) and 1 mg/500 ml mouse interferon-γ (Roche). Cells were maintained at 10% CO_2_ at 33°C.

### *In vitro* assays

For luciferase assays, H2K-*mdx* utrnA-luc cells were plated at 10 000 cells per well in a white solid tissue culture treated 96-well plate (BD Falcon). These were incubated in 10% CO_2_ at 33°C for 24 h prior to dosing. All compounds were supplied as a 10 mm solution in dimethyl sulfoxide (DMSO). Cells were treated with test compounds dissolved at a final concentration of 1% DMSO and assays performed in triplicate. Following 24 h incubation, luciferase levels were measured using the Luciferase assay system (Promega E1501) and plates read using a FLUOstar Optima plate reader (BMG Labtech). The mean was calculated from biological triplicates.

### Mice and drug treatment

Two-week-old male *mdx* (C57BL/10ScSn-Dmdmdx/J; Charles River Laboratories, MA, USA) littermates were randomly split and treated with SMT022357 (30 mg/kg) or vehicle only [phosphate buffered saline (PBS), 0.1% Tween-20, 5% DMSO] by daily intraperitoneal injection for 1 week and then daily oral gavage for a further 4 weeks. At the end of drug treatment, mice were sacrificed by CO_2_ asphyxiation in accordance with Schedule I of the UK Animals (Scientific Procedures) Act 1986 and muscle and blood samples taken.

### Blood analyses

Blood was collected with non-heparinized haematocrit tubes into serum microtainer tubes and centrifuged for 2 min at 12 000 rpm. Serum was stored at −80°C prior to analysis using the CK (NAC) reagent kit in conjunction with the AU 400 Clinical Chemistry analyser (Olympus UK Ltd).

### Histological analyses

Muscle samples were frozen in liquid nitrogen-chilled isopentane, and stored at −80°C. Transverse and longitudinal extensor digitorum longus (EDL), tibialis anterior (TA), soleus (SOL), diaphragm (DIA) and heart cryosections (10 μm) were stained with Haematoxylin and Eosin solution (H&E), Masson's trichrome (MT) and Alizarin red (AR). The Axioplan 2 Microscope System (Carl Zeiss, Germany) was used to obtain the images. The proportion of centrally nucleated fibres was determined by analysing the H&E images. Areas of necrosis were quantified based on the DMD_M.1.2.007 MDC1A_M.1.2.004 TREAT-NMD SOPS and performed with the Fiji ImageJ 1.49i software (73) on the whole EDL, SOL and DIA sections. Fibrosis of the whole DIA sections was observed using Masson's trichrome staining to visualize connective tissue and muscle fibres (Sigma kit HT15; SigmaAldrich) and quantified using a previously published macro (74) with the Fiji ImageJ 1.49i software.

### Immunofluorescence

Frozen transverse and longitudinal muscle sections were blocked in 10% foetal bovine serum/PBS for 20 min, incubated with primary antibodies for 1 h at room temperature or overnight at 4°C, washed in PBS and incubated with suitable Alexa Fluor secondary antibodies for 1 h at room temperature. Sections were examined under an Axioplan 2 Microscope System (Carl Zeiss, Germany). The following antibodies and dilution were used: goat polyclonal anti-utrophin (1:500, URD40) (75), mouse monoclonal anti- β-dystroglycan (1:100, MANDAG2, CIND), rabbit monoclonal anti- α1-CTFP/dystrobrevin (1:1000) (76), rabbit polyclonal anti desmin (1:100, ab8592, abcam), rabbit polyconal anti-collagen type I (1:100, 600-401-103-0.5, Rockland), mouse anti-MYHC1 (1:200, B8-F8), mouse anti-MYHC2A (1:200; SC-71); mouse IgM anti-MYHC2B (1:100, BF-F3; all from German Collection of Microorganisms and Cell Cultures) and polyclonal anti- α-Bungarotoxin AlexaFluo 488 conjugate antibody (1:500, B-13422, Life Technologies).

### Protein analyses

Western blots were performed according to standard procedures. Briefly, muscles were homogenized in Tris pH6.8 (75 mm), SDS (3.8%), Urea (4 m), glycerol (20%) supplemented with protease inhibitors (1:100; Sigma-Aldrich). Thirty micrograms of total protein were heat-denatured for 5 min at 100°C before loading onto NuPAGE 3–8% TRIS Acetate Midi Gel (Novex, Life Technlogies) and transferred to PVDF membranes (Millipore). Membranes were blocked for 1 h with 10% skimmed milk in 0.1% PBS-Tween20 and then incubated with primary antibodies in 0.1% PBS-T for 1 h at room temperature. Primary antibodies used were: mouse monoclonal anti-utrophin [1:50, MANCHO3(84A), gift from G.E. Morris], mouse monoclonal anti β-Dystroglycan (1:100, MANDAG2, CIND), rabbit monoclonal anti-α1-CTFP/dystrobrevin (1/1000), mouse monoclonal anti-Actin (1/1000, A2172, Sigma) and goat polyclonal α-actinin Antibody (N-19) (1:30 000, sc-7453, Santa Cruz). HRP-labelled secondary antibodies (Amersham) were incubated for 1 h at room temperature. Immunoreactive bands were detected using ECL Western Blot Detection Reagents (SuperSignal West Pico Chemiluminescent Substrate, Thermo Scientific). The relative expression of the target proteins was estimated by densitometry using Actin or Actinin N-19 as references on a Xograph Compact X4 developer and quantified with the Fiji ImageJ 1.49i software.

### RNA analyses

Cells were lysed in Buffer RLT and total RNA was extracted using the Qiagen RNeasy Kit (Qiagen) according to the manufacturer's instructions. 750 ng of total RNA was used to generate cDNA using the QuantiTect Reverse Transcription kit (Qiagen). Real-time PCR was performed on the StepOne™ Real-Time PCR system (Applied Biosystems) with TaqMan^®^ Fast Universal PCR Master Mix (Life Technologies). Results were analysed according to the Pfaffl method (77), a derivative of the ΔΔCT method (78). Utrophin (forward primer 5′-CGATGGACTCGCGTTCAAC-3′, reverse primer 5′ CCGGCACAAACCAGATC-3′) mRNA expression levels were normalized to the ‘normalization factor’ obtained from the geNorm software for Microsoft Excel 2010 which uses eukaryotic 18S rRNA (Catalog number: 4310893E, Life Technologies) and B2m (Assay number: Mm00437762_m1, Life Technologies) as reference genes (stability value <1.5). PCR amplification efficiency was determined by applying linear regression analysis to the exponential phase of the amplification curve of each PCR reaction using the LinRegPCR software (79). No reverse transcriptase (non-RT), no template control (NTC) reactions and non-contamination of cDNAs by genomic DNA (ALBh) were used as negative controls in each 40-cycle PCR run (Cq values NTC = undetermined, non-RT = undetermined and ALBh >35).

### Isolated muscle-function analysis

Peak force, specific force and force drop were measured from the extensor digitorum longus muscle of the treated and control mice. During dissection and experiments, muscles were bathed in oxygenated (95% O_2_–5% CO_2_) Krebs–Hensley solution composed of (mmol/l): NaCl, 118; NaHCO_3_, 24.8, KCl, 4.75; KH_2_PO_4_, 1.18; MgSO_4_, 1.18; CaCl_2_, 2.54; glucose, 10 (80). Contractile properties were measured as previously described (81). In brief, isolated EDL were attached to a lever arm connected to a force transducer (model 300B); and stimulator (model 701B); the equipment was controlled using the signal interface (model 604A) and results were recorded by the DMC software (version 4.1.4; Aurora Scientific, Aurora, Ontario, Canada). The muscle was stimulated by single pulses of 0.2 ms at 30 V while the optimum length (*L*_o_) was determined. Optimum fibre length (*L*_f_) was calculated by multiplying *L*_o_ by the predetermined fibre length to muscle length ratio of 0.44 (82). A force–frequency curve was generated and the maximum isometric force calculated. Absolute force (*P*_o_) are normalized to specific force (s*P*_o_; kN/m^2^) using the equation (muscle mass/L_f_ × 1.06 (the density of mammalian muscle). Percentage force drop were calculated by comparing maximum force between the first (ECC1) and fifth eccentric (ECC5) contractions, expressed as a percentage of ECC1. The muscle was stimulated into tetanus at the frequency required to generate the *P*_o_, while in tetanic state the muscle was stretched at a rate of one fibre length per second for 0.15 s, equating to a total stretch of 15% of fibre length. All data were digitized and analyzed using the DMA software (version 3.2; Aurora Scientific).

### Statistics

Results were analysed using Prism (GraphPad Software, Inc.) and the Student's *t*-test with a two-tailed distribution assuming equal or unequal sample variance depending of the equality of the variance (F-test). Data are presented as mean ± SEM (standard error of mean), with *n* indicating the number of independent biological replicates used in each group for comparison. Differences were considered significant at (*) *P* < 0.05; (**) *P* < 0.01 and (***) *P* < 0.001.

### Compounds

SMT022357 was provided by Summit Therapeutics plc. Structure and purity were confirmed by 1H NMR and LC-MS.

## Supplementary Material

Supplementary Material is available at *HMG* online.

## Funding

This work was supported by grants from the Medical Research Council, Muscular Dystrophy UK, the Muscular Dystrophy Association USA and Summit Therapeutics plc. Funding to pay the Open Access publication charges for this article was provided by the University of Oxford RCUK Open Access Block Grant.

## Supplementary Material

Supplementary Data
