# Supplementary material for: Second-generation compound for the modulation of utrophin in the therapy of DMD
Source: Hum Mol Genet. 2015 May 1;24(15):4212–24. doi: 10.1093/hmg/ddv154 (PMC4492389; doi:10.1093/hmg/ddv154)
Supplement: Supplementary Data [file supp_24_15_4212__index.html]

Second-generation compound for the modulation of utrophin in the therapy of DMD — Second-generation compound for the modulation of utrophin in the therapy of DMD — Second-generation compound for the modulation of utrophin in the therapy of DMD — Supplementary Data 

# Second-generation compound for the modulation of utrophin in the therapy of DMD

## Supplementary Data

Supplementary Data

**Files in this Data Supplement:**

- Supplementary Data - pptx file
