# Supplementary material for: Second-generation compound for the modulation of utrophin in the therapy of DMD
Source: Hum Mol Genet. 2015 May 1;24(15):4212–24. doi: 10.1093/hmg/ddv154 (PMC4492389; doi:10.1093/hmg/ddv154)
Supplement: Supplementary Data [file supp_ddv154_ddv154supp.pptx]

## Slide 1
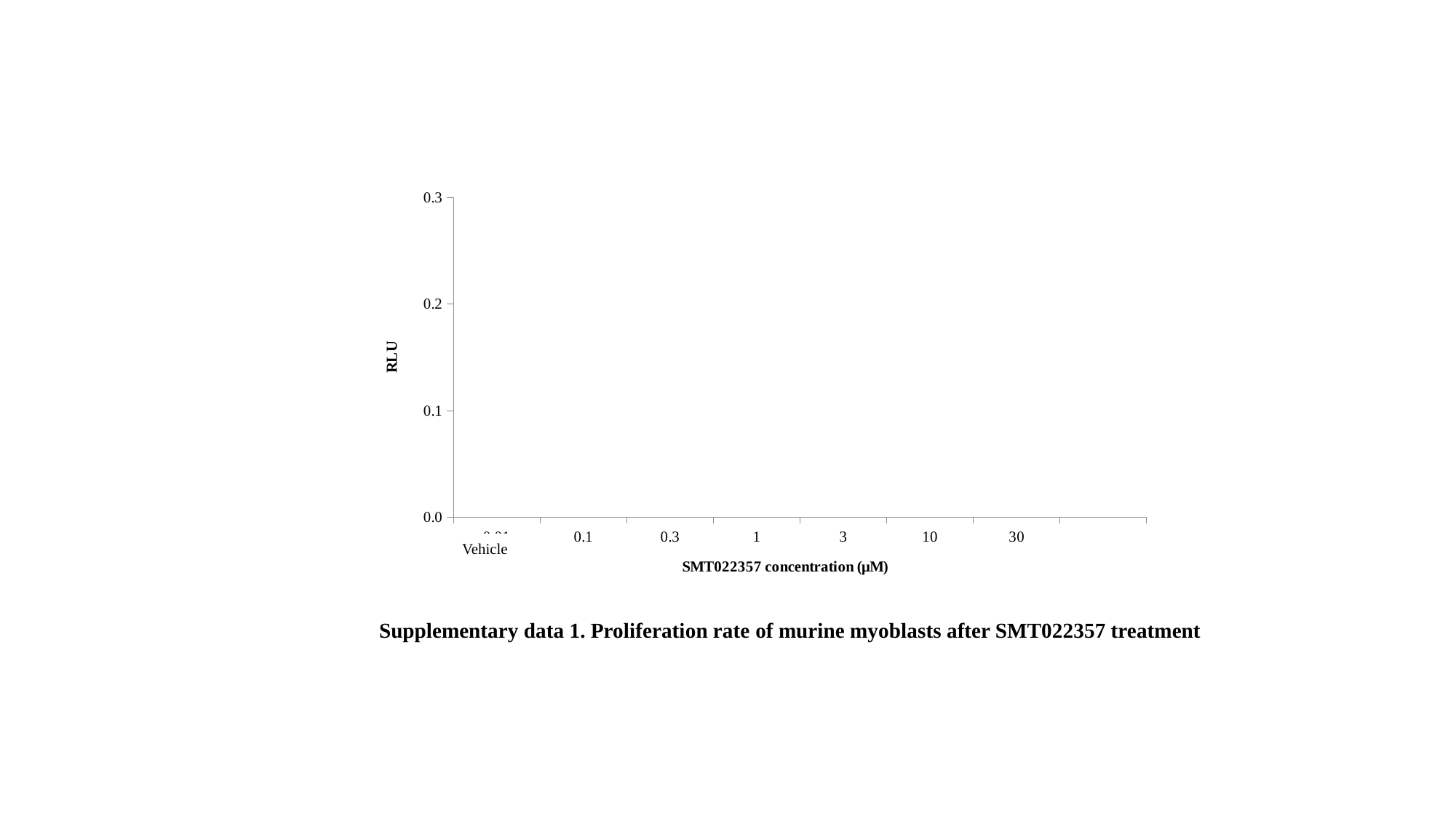

### Chart
| Category | SMT022357 |
|---|---|
| 0.01 | 0.2502666666666667 |
| 0.1 | 0.2627333333333333 |
| 0.3 | 0.2656 |
| 1 | 0.2602 |
| 3 | 0.2589666666666667 |
| 10 | 0.2571 |
| 30 | 0.25696666666666673 |Vehicle
Supplementary data 1. Proliferation rate of murine myoblasts after SMT022357 treatment

## Slide 2
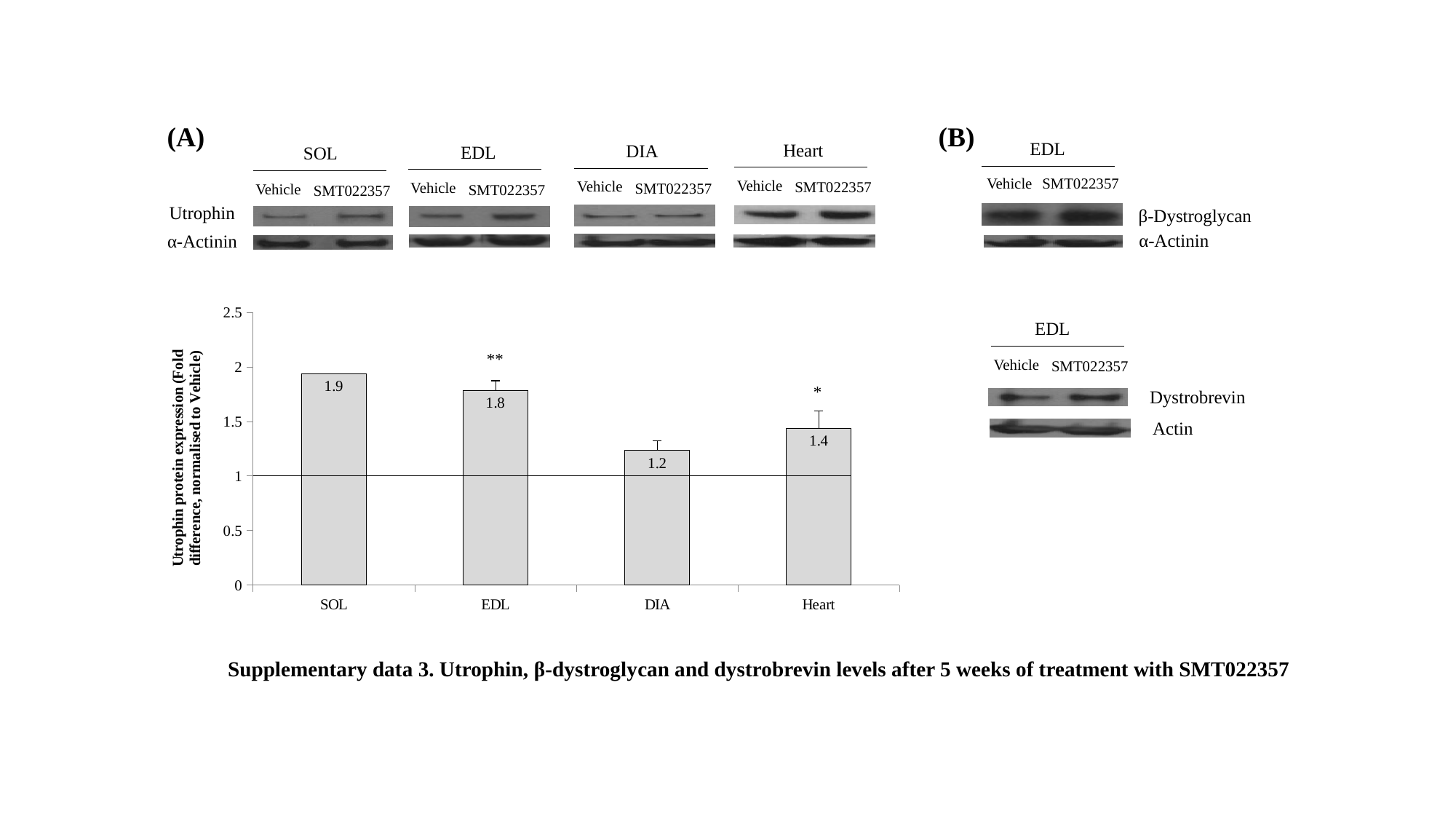

(A)
(B)
EDL
Heart
DIA
EDL
SOL
SMT022357
Vehicle
Vehicle
Vehicle
SMT022357
Vehicle
SMT022357
Vehicle
SMT022357
SMT022357
Utrophin
β-Dystroglycan
α-Actinin
α-Actinin
### Chart
| Category | |
|---|---|
| SOL | 1.94 |
| EDL | 1.7867462110802592 |
| DIA | 1.2335333022228325 |
| Heart | 1.438936858330617 |EDL
**
Vehicle
SMT022357
*
Dystrobrevin
Actin
Supplementary data 3. Utrophin, β-dystroglycan and dystrobrevin levels after 5 weeks of treatment with SMT022357
